# Urinary markers of *Mycobacterium tuberculosis* and dysbiosis in paediatric tuberculous meningitis cases undergoing treatment

**DOI:** 10.1186/s13099-024-00609-9

**Published:** 2024-03-12

**Authors:** Simon Isaiah, Du Toit Loots, A. Marceline Tutu van Furth, Elmarie Davoren, Sabine van Elsland, Regan Solomons, Martijn van der Kuip, Shayne Mason

**Affiliations:** 1 Human Metabolomics, Faculty of Natural and Agricultural Sciences, North-West University, Potchefstroom, South Africa; 2 Vrije Universiteit, Pediatric Infectious Diseases and Immunology, Amsterdam University Medical Centers, Emma Children’s Hospital, De Boelelaan 1117, Amsterdam, The Netherlands; 3 Centre for Human Metabolomics, North-West University, Potchefstroom, South Africa; 4 MRC Centre for Global Infectious Disease Analysis, Imperial College London, London, UK; 5 Department of Paediatrics and Child Health, Faculty of Medicine and Health Sciences, Stellenbosch University, Cape Town, South Africa

**Keywords:** Urine, Microbiota, Paediatric, Tuberculous meningitis (TBM), Metabolomics, Liquid chromatography-tandem mass spectrometry (LC-MS/MS)

## Abstract

**Background:**

The pathogenesis of tuberculous meningitis (TBM) involves infection by *Mycobacterium tuberculosis* in the meninges and brain. However, recent studies have shown that the immune response and inflammatory processes triggered by TBM can have significant effects on gut microbiota. Disruptions in the gut microbiome have been linked to various systemic consequences, including altered immunity and metabolic dysregulation. Inflammation caused by TBM, antibiotic treatment, and changes in host immunity can all influence the composition of gut microbes. This complex relationship between TBM and the gut microbiome is of great importance in clinical settings. To gain a deeper understanding of the intricate interactions between TBM and the gut microbiome, we report innovative insights into the development of the disease in response to treatment. Ultimately, this could lead to improved outcomes, management strategies and quality of life for individuals affected by TBM.

**Method:**

We used a targeted liquid chromatography–tandem mass spectrometry (LC-MS/MS) approach to investigate metabolites associated with gut metabolism in paediatric participants by analysing the urine samples collected from a control group (*n* = 40), and an experimental group (*n* = 35) with confirmed TBM, which were subdivided into TBM stage 1 (*n* = 8), stage 2 (*n* = 11) and stage 3 (*n* = 16).

**Findings:**

Our metabolomics investigation showed that, of the 78 initially selected compounds of microbiome origin, eight unique urinary metabolites were identified: 2-methylbutyrlglycine, 3-hydroxypropionic acid, 3-methylcrotonylglycine, 4-hydroxyhippuric acid, 5-hydroxyindoleacetic acid, 5-hydroxyhexanoic acid, isobutyrylglycine, and phenylacetylglutamine as urinary markers of dysbiosis in TBM.

**Conclusion:**

These results – which are supported by previous urinary studies of tuberculosis – highlight the importance of gut metabolism and of identifying corresponding microbial metabolites as novel points for the foundation of improved management of TBM patients.

**Supplementary Information:**

The online version contains supplementary material available at 10.1186/s13099-024-00609-9.

## Introduction

Before the coronavirus (COVID-19) pandemic, tuberculosis (TB) was ranked as the leading cause of infectious disease mortalities worldwide [1, 2]. Tuberculous meningitis (TBM) is a severe form of extrapulmonary TB, resulting in high morbidity and mortality, especially in children under the age of 12 years [3]. Compared to that of pulmonary TB, less research has been conducted on TBM, mainly due to difficulty in attaining research sample material, particularlycerebrospinal fluid, comparatively lower prevalence of TBM and difficulty in diagnosis. Hence, much still needs to be done to better understand *Mycobacterium tuberculosis* (*M. tb*), the causative agent, in the context of TBM, the subsequent immunopathological interactions and the resulting systemic metabolic adaptations/response of the host, which in turn typically leads to new therapeutic approaches [2–7]. There is also an urgent need for more effective and earlier diagnostic tools to detect TBM, especially in children, at an early stage of the disease.

The human gut microbiome is a complex dynamic ecosystem. Dysbiosis – disruption of the gut microbiota – has been found to be strongly correlated with many infectious diseases, including TB and TBM. Mason et al. [8] previously identified four urinary metabolites – methylcitric, 2-ketoglutaric, quinolinic and 4-hydroxyhippuric acids, ascribed to an altered host microbiome in children with TBM. Zhou et al. [9] observed that the microbiota of both intra- andextra-TB lesions are similar, and identified Mycobacteria and Porphyromonas as co-factors for lesion formation. Krishna et al. [10] reported significant changes to the microbial composition of the lung microbiome of pulmonary TB patients vs. control groups, and also different opportunistic pathogens characterized the *M. tb-*infected cases. Isaiah et al. [11] additionally identified 15 urinary metabolites characterising advanced stage TBM, resulting from six dysregulated metabolic pathways and a disrupted gut microbiome. Luies et al. [12] identified 50 urinary metabolite markers characterising pulmonary TB, as well as investigated successful and unsuccessful treatment outcomes of standard anti-TB therapy, with one of the main reasons for unsuccessful treatment outcome being an altered gut microbiome.

Metabolomics is a modern scientific approach, based upon using the latest analytical and bioinformatics methods, and capable of accurate quantitative characterization of metabolites [13]. The discipline has resulted in a substantial and rapid advancement of knowledge by identifying new biomarkers associated with various diseases, and metabolomics research into *M. tb* infection is no exception [14–16]. As described, various untargeted metabolomics studies have identified dysbiosis as an important component of *M. tb* infection. We postulate that a targeted microbiome metabolite metabolomics study, using urine samples collected from patients with confirmed TBM during the 6 month treatment regimen, would show changes in microbiome metabolites associated with: (1) the gut microbiome, (2) host–microbe interaction, and also possibly (3) *M. tb* death – identifiable by metabolites associated with the *M. tb* cell wall. Thus, this study aimed to identify urinary metabolites associated with host and/or microbial changes associated with dysbiosis in TBM cases during the course of treatment.

## Materials and methods

### Sampling

The sampling cohort in this study were from a population of infants and children (< 13 years) living in the Western Cape province of South Africa, a region well known for a high prevalence of TB [17–19]. Paediatric patients from primary and secondary healthcare service centres were referred to the paediatric department at the Tygerberg Hospital in Cape Town. Written and informed consent or assent was obtained from all participants.

The urine samples collected from the participants were divided into two groups: Group 1, the controls (*n* = 40) and Group 2, confirmed TBM (*n* = 35), which were subdivided into TBM stage 1 (*n* = 8), stage 2 (*n* = 11) and stage 3 (*n* = 16), as described by van Toorn et al. [20]. The controls (Group 1) were age-matched and collected from the same geographical region as the TBM paediatric patients. They were seen at the general paediatrics outpatient clinic of Tygerberg Hospital for follow up of an underlying subacute or chronic illness. None of them had meningitis or neurological disease during sampling. The TBM urine samples (Group 2) were collected from paediatric cases admitted to Tygerberg Hospital due to suspected meningitis, most of whom were seriously ill, and later confirmed to be “definite TBM”, identified by the presence of *M. tb* in their cerebrospinal fluid by microscopy, culture, and/or detection by nucleic-acid techniques, with or without a focal neurological deficit [21, 22]. The TBM patients were stabilized before hospital discharge (the median time from admission to discharge in the study setting over the past 38 years was 16 days, interquartile range 12–23 days) and a baseline urine sample was collected upon initiation of home treatment, as described by [23]. Ethical approval (numbers N16/11/142 and N11/03/061 for groups 1 and 2, respectively), was obtained by the Health Research Ethics Committee (HREC) at Stellenbosch University in accordance with the Western Cape provincial government policy and the HREC at North-West University, Potchefstroom campus (ethics approval number NWU-00063-18-A1-01). All participants with a confirmed positive HIV status were excluded from this study, since HIV co-infection would significantly complicate an already complex metabolic profile. Supplementary Table S1 shows demographic information of study cohort.

### Sample handling and storage

After collection, all urine samples were stored at − 80 °C in the Molecular Biology and Human Genetics Division of the University of Stellenbosch. All samples were transported overnight on dry ice and stored in a designated freezer (–80 °C) in a biosafety level 3 (BSL-3) laboratory at North-West University, Potchefstroom campus. Initially, all urine samples were thawed in a biosafety cabinet and 100 µL of each sample (TBM and controls) was aliquoted into a single tube to create a pooled urine quality control sample (QC). A separate pooled urine sample consisting of 100 µL aliquots from the TBM cases only was also prepared (PS). Each pooled sample (QC and PS) was then vortexed and equal aliquots of each sample were placed in Eppendorf microcentrifuge tubes. These two types of quality control samples were used to monitor the analytical performance during the duration of the experiment. All samples (experimental, QC and PS) were stored again at − 80 °C.

### Chemicals and standards

All mobile phase solutions were prepared with UPLC–MS grade solvents of water and acetonitrile from Anatech (Burdick and Jackson), and formic acid was obtained from Merck (South Africa). Supplementary Table S3 shows the 78 organic acid standards used in this study, and their source. NOTE: these specific 78 organic compounds were selected for this targeted study because they are considered to be part of gut microbial metabolism.

### Sample preparation and LC–MS/MS analysis

Urine samples were thawed at room temperature and homogenized by vortex mixing. A 50 µL volume of urine was aliquoted into a microcentrifuge tube and to each sample, 50 µL of internal standard (heptanoylglycine: 576.8 µM) and 200 µL of HPLC water with 0.1% formic acid were added, and the sample vortexed. A 200 µL volume of the prepared sample was then transferred into a 2 mL glass vial containing an insert and capped. The samples were analysed (in 19 batches of 12–14 samples interspaced with QC and PS samples) on an Infinity II 1290 UHPLC–MS/MS system (Agilent Technologies, Chemetrix, South Africa). Prior to sample analysis, Mobile phase (A and B) standard solutions were freshly prepared: (A) HPLC water with 0.1% v/v formic acid, (B) acetonitrile with 0.1% v/v formic acid. The temperature within the multi-sampler was set at 4°C and the column temperature at 22°C. The flow rate was set at 0.4 mL/min, sample injection at 2 µL and the run time was 27 min. LC-separation was accomplished using an Acquity C18 column UPLC HSS T3 1.8 μm (2.1 × 100 mm) with an Acquity VanGuard Pre-Column HSS T3, 2.1 × 5 mm (Waters, South Africa). The gradient programme of the 27 min run in negative ionization mode and the MS conditions are described in Supplementary Table S5. The PS were randomized across the batches with QCs placed at the start, middle and end of each batch run. All QC and PS extracts were injected twice.

Data acquisition and processing was conducted using Agilent MassHunter workstation software, specifically Acquisition V10 and Quantitative Analysis for QQQ V10 Software. The monitored ions and their retention times, which were determined using standards, are listed in Table S4. The peak area was used for quantification and each analyte quantified relative to the internal standard. A data matrix was generated in Excel that consisted of all cases as rows, and the 78 metabolites as columns, with each entry representing a relative concentration.

### Statistical analysis

For all multivariate statistical analyses, a log transformation of the data was conducted to correct for data distribution (i.e., Gaussian distribution; parametric data) and heteroscedasticity, and auto scaling was applied to allow different values with differences in magnitudes of order (concentrations) to be more comparable. All univariate statistical analyses were done on the non-parametric (untransformed) data. MetaboAnalyst 5.0 (www.metaboanalyst.ca) was used to provide an initial qualitative visualization of the distribution and variation of the data at a multivariate level by using unsupervised principal component analysis (PCA). MetaboAnalyst was also used for the multivariate statistical analyses of the data using supervised partial least squares discriminant analysis (PLS–DA) – which was tested for overfitting by conducting a permutation test – and univariate assessment of the data by a one-way ANOVA (Kruskal–Wallis test) with a statistical significance set at a p-value cut-off at a false discovery rate (FDR) less than 0.01. The PLS–DA model was used quantitatively to identify significant metabolites as having variables important in projection (VIP) values > 1.0 for components 1 and 2. Additional univariate statistics was conducted using the non-parametric equivalent of a one-way ANOVA, with a significant adjusted p-value < 0.01. Microsoft Excel was used to calculate effect size, in order to determine practical significance. The most important metabolites, best describing the variation between the compared groups, were identified using the following criteria: PLS–DA VIP value (components 1 and 2) > 1.0 and ANOVA Kruskal–Wallis FDR p-value < 0.01 and effect size d-value ≥ 0.8. GraphPad Prism (V 10.0.2) was used to create violin plots of the metabolites identified as important – illustrating distribution of data frequency, medians, and interquartile ranges, and statistically significant differences (with multiple corrections) noted as ANOVA Kruskal–Wallis FDR p-value < 0.01, as well as linear regression plots.

## Results

### Quality assurance

PCA of all the batches (*n* = 19) was conducted to determine if there were any batch effects. Notably, one case (014_T3) stood out as an outlier (see supplementary information (SI) – Fig. S1) and was removed because this was a highly diluted sample (creatinine concentration = 0.026 mM) and normalizing this case to creatinine overinflated all other values. Reassessment of the PCA of all batches after removal of this single outlier case showed no batch effects, therefore batch correction was not necessary. An assessment of the PCA (Fig. S2) showed that all the QC and PS samples clustered closely together, confirming that there were no batch effects in the data, supporting the data for further statistical analyses and metabolite marker selection. An additional quantitative quality check was done on all the QC samples by calculating the coefficient of variation (CV) of each, and across each batch. Of the 78 metabolites analysed, three metabolites had an overall CV greater than 20%, namely, propionylglycine (55.06%), suberylglycine (38.7%), and 3,4-dihydroxybenzoic acid (22.41%), which were subsequently removed from the metabolite list. Hence, quality assurance checks of the data removed one TBM case and three metabolites, yielding 75 metabolites suitable for analysis and biological interpretation.

### Initial urinary profile

**Fig. 1:**
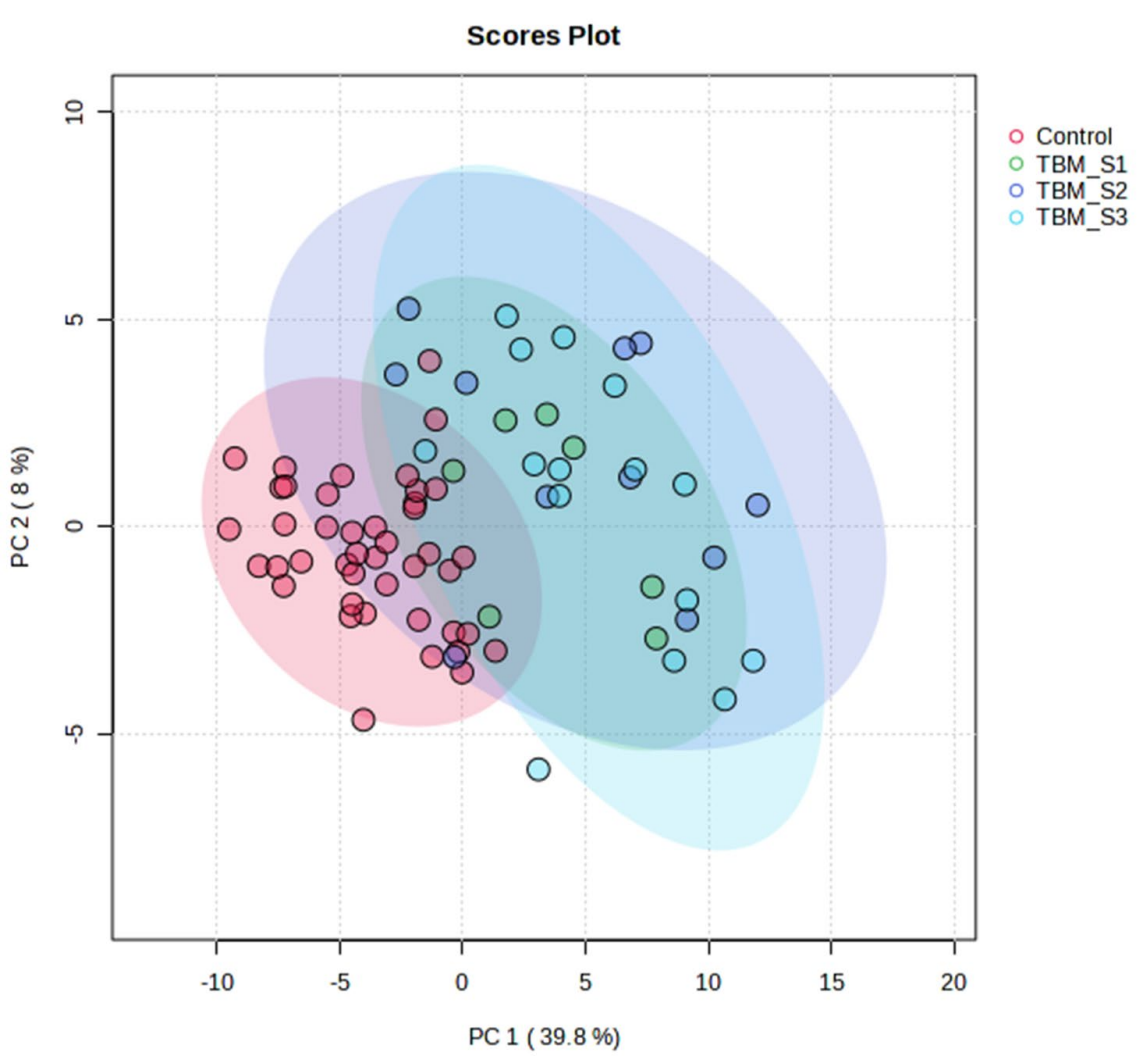
PCA scores plot of initial urinary profile for all 75 metabolites analysed at T1. PCA 1 & 2 explained 48% of the total variation

The first urine sample collected from each TBM participant after discharge from hospital was labelled “T1” to represent the initial urine sample. There were initially 7, 11 and 16 urine samples representing TBM stages 1, 2 and 3, respectively. The PCA indicated that the metabolome of the control group differentiated significantly from the TBM cases, with PCA 1 & 2 explaining 48% of the total variation (Fig. 1). The natural separation in the PCA between the control group and TBM cases provided confidence in using PLS–DA modelling. The PLS–DA model generated (Fig. S3) is also supported by a permutation p-value of 0.001 (Fig. S4), thus the data were not over-fitted in the PLS–DA model. Quantitative data indicating 30 metabolites at T1 that are of statistical and practical importance are shown in Table S2. No differentiation between TBM stages at T1 could be seen based upon PCA (Fig. S3).

### Longitudinal urinary profile during TBM treatment

**Fig. 2:**
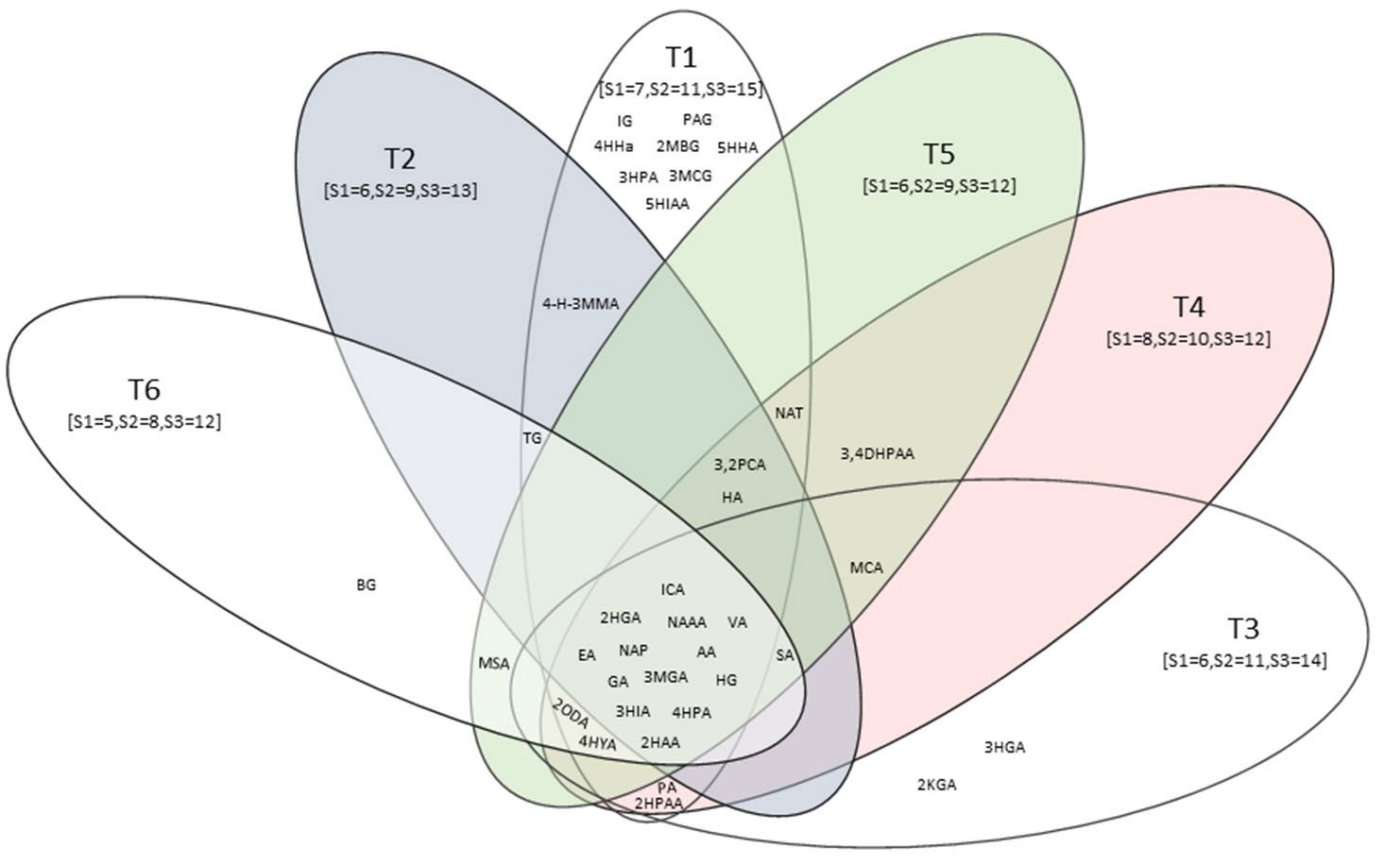
Venn diagram of significant metabolites across all six time points of TBM treatment. Number of cases according to TBM stages per time point are given in square brackets. Abbreviations: 2HGA, 2-hydroxyglutaric acid; 3HIA, 3-hydroxyisovaleric acid; 3MGA, 3-methylglutaconic acid; 4HPAA:4-hydroxyphenylacetic acid; AA, aconitic acid; EA, ethylmalonic acid; GA, glucaric acid; HG, hexanoylglycine; ICA, isocitric acid; NAAA, N-acetylaspartic acid; NAP, N-acetylphenylalanine; VA, vanillactic acid; 2HAA, 2-hydroxyadipic acid; 2HPAA, 2-hydroxyphenylacetic acid; 2KGA, 2-ketoglutaric acid; 2MBG, 2-methylbutyrylglycine; 2ODA, 2-octenedioic acid; 2,3PCA, 2,3-pyridinecarboxylic acid; 3HGA, 3-hydroxyglutaric acid; 3HPA, 3-hydroxypropionic acid; 3MCG, 3-methylcrotonylglycine; 3,4DHPAA, 3,4-dihydroxyphenylacetic acid; 4-H-3MMA, 4-hydroxy-3-methoxymandelic acid; 4HPLA, 4-hydroxyphenyllactic acid; 4HHA, 4-hydroxyhippuricacid; 5HIAA, 5-hydroxyindoleacetic acid; 5HHA, 5-hydroxyhexanoic acid; BG, buterylglycine; HA, homovanillic acid; IG, isobutyrylglycine; MCA, methylcitric acid; MSA, methylsuccinic acid; NAT, N acetyltyrosine; PAG, phenylacetylglutamine; PA, pimelic acid; SA, suberic acid; TG, tiglylglycine

The same statistical approach as described for the initial urinary profile comparisons was adopted for time points 2 to 6 (T2–T6), for the samples collected monthly during the 6-month treatment regimen. Hence, T2 was one month after the initial sample was collected and represented the altered metabolome after one month of treatment; T6 was collected five months after the initial sample and represented five months of treatment. It is important to note that not all TBM cases had 100% adherence to IZN, PYR and RIF throughout the entire treatment period. By T4, there was a decrease in adherence for IZN: 100–89.7%; PYR: 100–95.2%; and RIF: 65.5–34.5%; although, RIF adherence declined over time apparently due to its bad taste [23]. For all longitudinal time points (T2–T6), the PCAs similarly reflected (see SI) some TBM cases beginning to cluster closer to controls; however, a clear differentiation between the control group and all three TBM stages remained. Similarly to the results of the initial urinary profile comparison, the PLS–DA model remained reliable (permutation p-value < 0.05). Following the same rule described for the initial urinary profile comparison, five additional data sets were generated (see SI). A Venn graph of the common and unique metabolites for all six time points is shown in Fig. 2. Furthermore, similarly to T1 (Fig. S3), TBM stages did not differentiate from each other across the longitudinal comparison of the study.

**Fig. 3:**
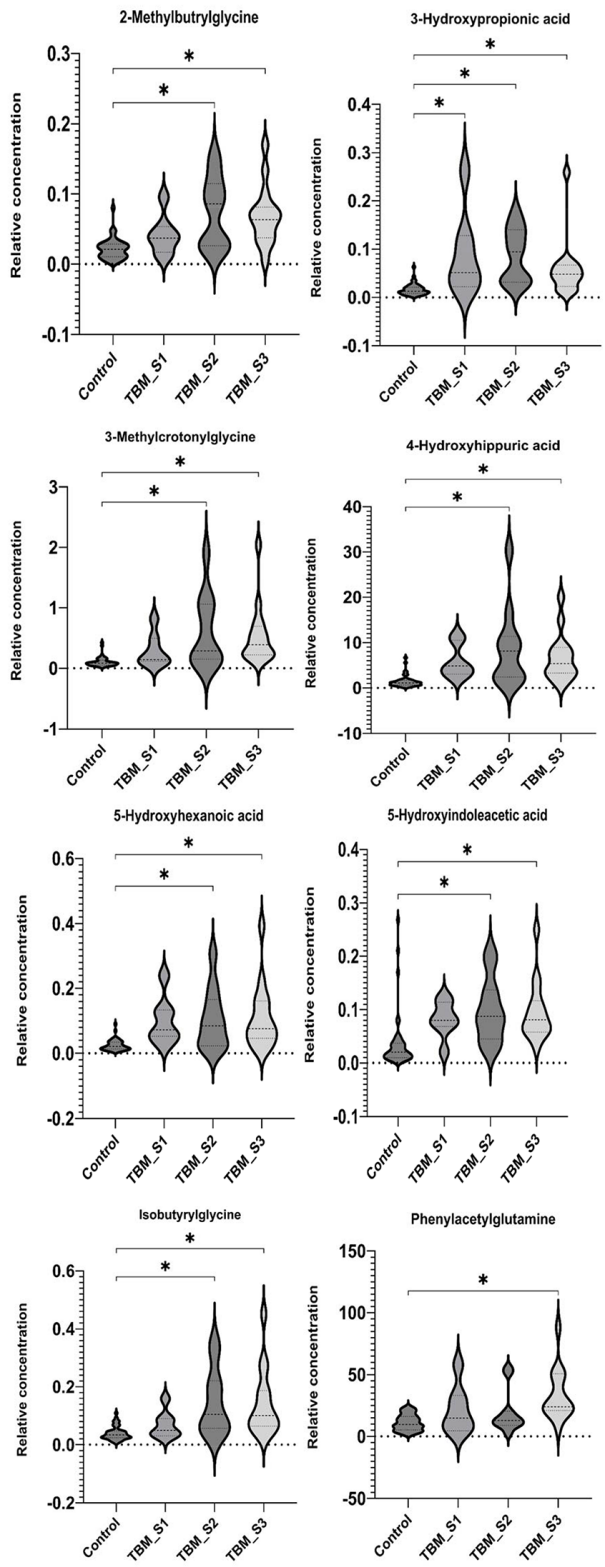
Violin plots of eight significant metabolites at T1: 2-methylbutyrlglycine, 3-hydroxypropionic acid, 3-methylcrotonylglycine, 4-hydroxyhippuric acid, 5-hydroxyindoleacetic acid, 5-hydroxyhexanoic acid, isobutyrylglycine, and phenylacetylglutamine. Control, TBM S1, TBM S2 and TBM S3 consist of 40, 7, 11 and 15 number of samples, respectively. Plots show distribution of data frequency, as well as the median and interquartile ranges. Statistically significant differences (ANOVA Kruskal–Wallis FDR *p* < 0.01, with multiple corrections) indicated by *

**Fig. 4:**
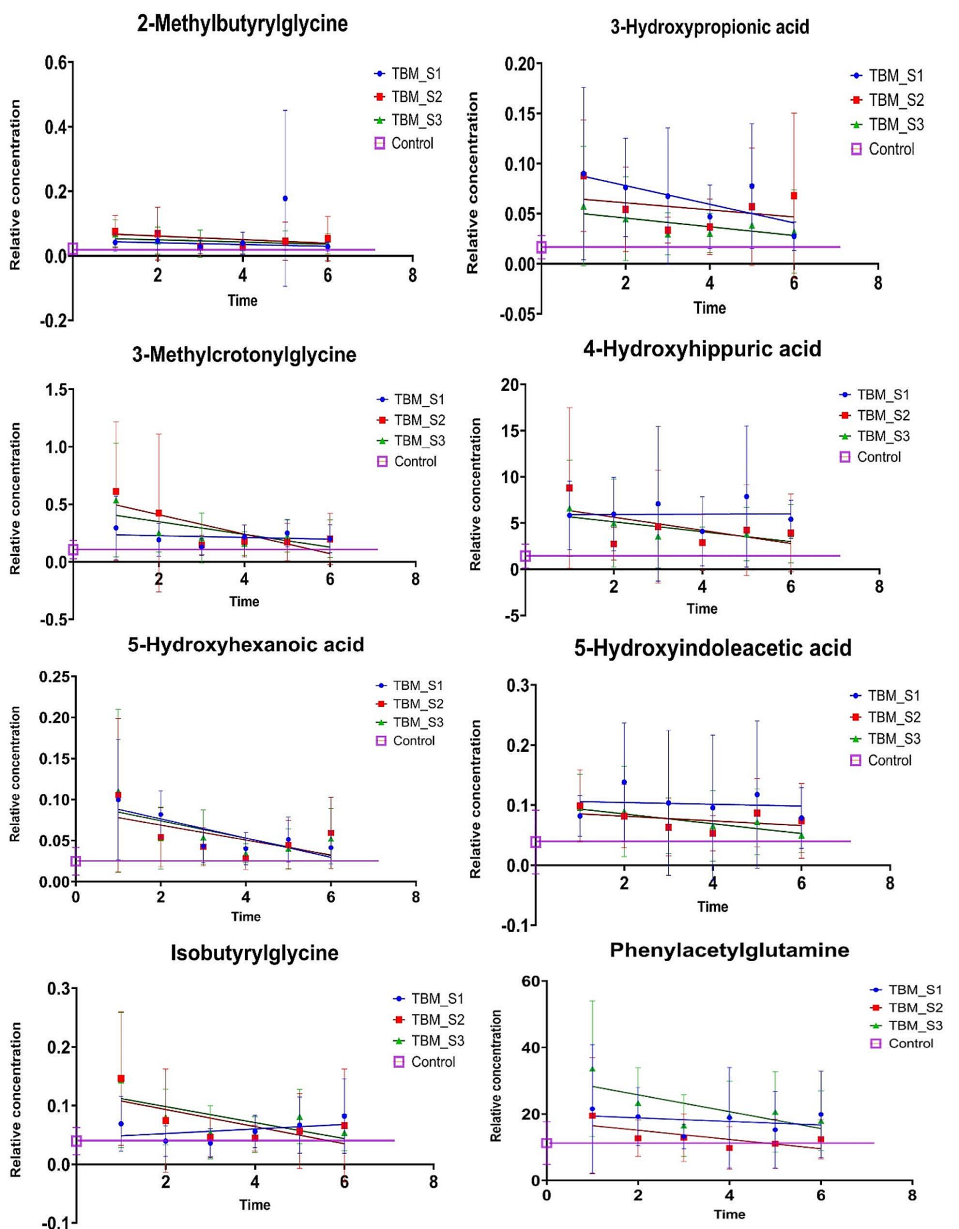
Linear regression plots with error plots across all six TBM treatment points (T1–T6), for control, TBM_S1, TBM_S2 and TBM_S3, of eight metabolites found to be significant only at T1. All eight metabolites show increased levels at T1, returning to normal (control) levels at final treatment point (T6) for all S2 and S3, while some metabolites remain consistently elevated for S1. Metabolites: 2-methylbutyrlglycine, 3-hydroxypropionic acid, 3-methylcrotonylglycine, 4-hydroxyhippuric acid, 5-hydroxyindoleacetic acid, 5-hydroxyhexanoic acid, isobutyrylglycine, and phenylacetylglutamine

Figure 2 shows that eight significant metabolites (2-methylbutyrlglycine, 3-hydroxypropionic acid, 3-methylcrotonylglycine, 4-hydroxyhippuric acid, 5-hydroxyindoleacetic acid, 5-hydroxyhexanoic acid, isobutyrylglycine, and phenylacetylglutamine – see violin plots in Fig. 3 and linear regression plots in Fig. 4) uniquely occur only in T1. Since these eight metabolites occur exclusively in the T1 samples (not significant in T2–T6), they represent the metabolites induced by TBM disease and hence will be interpreted in this context. Other significant metabolites that occurred uniquely at the other time points were butyrylglycine at T6, and 2-ketoglutatric acid and 3-hydroxyglutaric acid at T3. The 13 significant metabolites common across all time points included: 2-hydroxyadipic acid, 2-hydroxyglutaric acid, 3-hydroxyisovaleric acid, 3-methylglutaconic acid, 4-hydroxyphenylacetic acid, aconitic acid, ethylmalonic acid, glucaric acid, hexanoylglycine, isocitric acid, N-acetylaspartic acid, N-acetylphenylalanine, and vanillactic acid. Since these 13 metabolites were common across all six treatment time points, they are markers of treatment and will be discussed further in this context. Other significant metabolites that occurred commonly at some, but not all, time points were: 2-hydroxyphenylacetic acid at T1, T3 and T4; 2-octenedioic acid at T1, T3, T4, T5 and T6; 2,3-pyridinecarboxylic acid (quinolinic acid) at T1, T2, T4 and T5; 3,4-dihydroxyphenylacetic acid at T4 and T5, 4-hydroxy-3-methoxymandelic acid at T1 and T2; 4-hydroxyphenyllactic acid at T1, T3,T4,T5 and T6; homovanillic acid at T1, T2, T4 and T5; methylcitric acid at T3, T4 and T5; methylsuccinic acid at T5 and T6, N-acetyltyrosine at T1, T4 and T5; pimelic acid at T3 and T4; suberic acid at T2, T3, T4, T5 and T6; and tiglylglycine at T1, T2 and T6.

## Discussion

In this targeted LC–MS/MS metabolomics investigation, of initially 78 selected compounds of microbiome origin, we identified eight unique, statistically significant urinary metabolites at T1: 2-methylbutyrlglycine (2MBG), 3-hydroxypropionic acid (3HPA), 3-methylcrotonylglycine (3MCG), 4-hydroxyhippuric acid (4HHA), 5-hydroxyindoleacetic acid (5HIAA), 5-hydroxyhexanoic acid (5HHA), isobutyrylglycine (IG), and phenylacetylglutamine (PAG). Five of these eight significant metabolites (2MBG, 3MCG, 4HHA, 5HIAA, and PAG) showed a constant elevated level for TBM stage 1 (Fig. 4) across the entire treatment period and we consider this to be the background effect of the treatment. We consider these eight metabolites to be associated with the disease pathogenesis (TBM), as markers of the breakdown of *M. tb* cell wall caused by anti-TB medication, and/or dysbiosis commonly associated TBM. We discuss the biological significance of these eight metabolites in terms of: (1) associated metabolic pathways and corroboration from the literature; (2) their origin – linking these metabolites to either microbial or host origin, or a combination of both (a co-metabolite); (3) the insight(s) into the biology of the breakdown of *M. tb* cell wall within the host; (4) understanding the gut dysbiosis associated with TBM.

### Metabolic map and link to previous TB marker studies

**Fig. 5:**
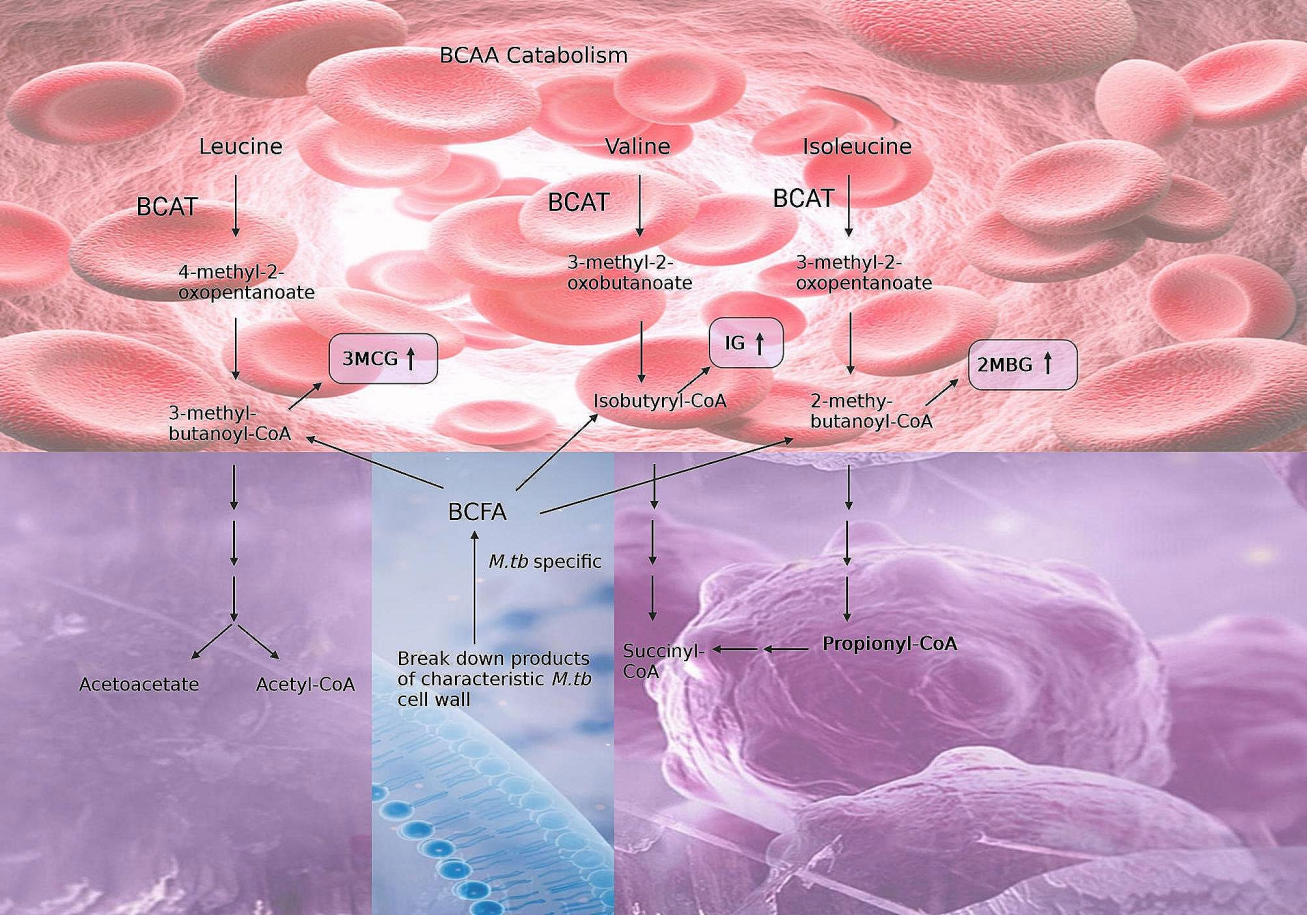
Branched-chain amino acid (BCAA) catabolism (in the host: red; and as co-metabolism (both host and microbial): purple). Elevated 3-methylcrotonylglycine (3MCG), isobutyrylglycine (IG), and 2-methylbutyrylglycine (2MBG) are urinary markers of dysbiosis at T1 of TBM treatment. It should be noted that the three BCAA intermediates (3-methylbutaonyl-CoA, isobutyrl-CoA and 2-methylbutanoly-CoA) can also be linked to branched-chain fatty acids that are specific to deterioration of the unique cell wall of *M. tb* (microbial specific: blue). Propionyl-CoA is also an end product that can be linked to Fig. 6

It should be noted that seven of the eight significant metabolites identified in this study have previously been linked to pulmonary TB. IG, 3MCG and 2MBG are glycine conjugates of intermediates (isobutyryl-CoA, 3-methylcrotonyl-CoA and 2-methylbutyryl-CoA, respectively) of branched-chain amino acid catabolism (Fig. 5). Based upon a transcriptomics study by Jiang et al. [24], the expression of the enzyme branched-chain amino acid transferase-1 (BCAT1) is decreased, whereas BCAT2 increases, in TB cases. Both BCAT1 and BCAT2 are of human origin, with BCAT1 being localized in the cytoplasm and BCAT2 compartmentalized in the mitochondria. An increased expression of (mitochondrial) BCAT2 is in line with the enhanced energy metabolism associated with TB [8]. However, raised BCAT2 activity is not specific to TB only, but has also been linked to other inflammatory diseases [25]. Furthermore, propionyl-CoA is an end product of valine and isoleucine catabolism, and is a microbial metabolite, as discussed later.

Four of the eight significant metabolites – 3HPA, 5HIAA, 5HHA, and PAG – can also be linked to urinary markers of pulmonary TB as part of the altered host metabolome, caused by *M. tb* [26]. 3HPA is a microbial product of glycerol [27], a fatty acid that was identified as increased in TB cases by Luier and Loots [26]. 5HHA (a fatty acid) and 5HIAA (a tryptophan catabolite) were found to be increased in our study and also by Luier and Loots [26]. PAG, increased in this study, is a biotransformation product of phenylacetic acid, and also found to be increased by Luier and Loots [26].

### 3-Hydroxypropionic acid (3HPA) – host immune response, gut microbiome and *M. tb* metabolism

**Fig. 6:**
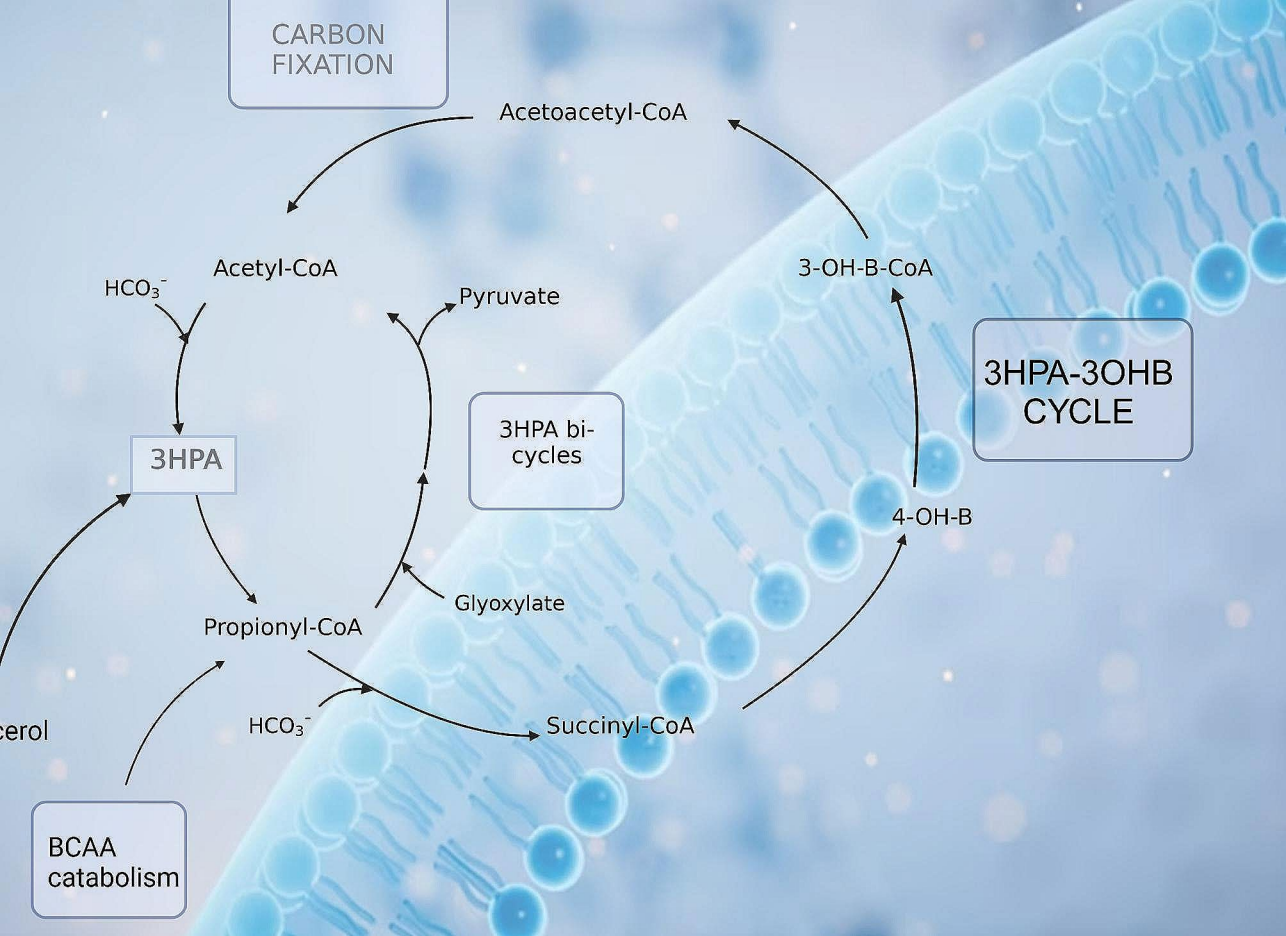
Microbial-specific (blue background) bi-cycle of acetyl-CoA, propionyl-CoA and carbon fixation via the microbial cycle of hydroxypropionic acid and hydroxybutyric acid. Both these microbial cycles rely upon the intermediary 3HPA, which is elevated and associated with dysbiosis during TBM.

The metabolite 3HPA was the only metabolite that was significantly (adjusted *p* < 0.01) increased in all three TBM stages at T1 (Fig. 3). Treatment of TBM stages 1 and 3 (Fig. 4) showed 3HPA returning to that of the control levels by T6. TBM stage 2 showed a similar linear trend as stages 1 and 3, up to T4; thereafter, there was a re-emergence of increased 3HPA. Elevated levels of 3HPA in the host usually can be caused by defect in the enzyme propionyl-CoA carboxylase. Certain metabolic conditions in the host have been linked to appearance of high concentration of 3HPA in urine. These include: propionyl-CoA carboxylase deficiency, combined carboxylase deficiencies resulting from a defect of biotin metabolism, methylmalonic semialdehyde dehydrogenase deficiency, and 4-hydroxybutyric aciduria [28–31]. Significant amounts of 3HPA can bring about functioning as an acidogen (induce metabolic acidiosis) and metabotoxin (prompts adverse effect at chronic high levels) [32, 33]. In a study reported by Pollitt et al. [29], three infants died due to a peculiar pattern of a significant urinary excretion of 3HPA, attributed to abnormal bacterial metabolism in the gut. 3HPA is an intermediary in the degradation of gut-produced propionic acid and branched-chain amino acids, as well as an intermediary metabolite in the microbial bi-cycle of propionyl-CoA and acetyl-CoA, and for carbon fixation via the microbial cycle of hydroxypropionic acid and hydroxybutyric acid (Fig. 6). Furthermore, 3HPA is a key intermediate in propionyl-CoA metabolism, which is used by *M. tb* via three mechanisms: (1) the protein ISL1 converts propionyl-CoA to succinic acid and pyruvic acid for energy production via either isocitrate lyase in the glyoxylate shunt or methylisocitrate lyase in the methylcitric acid cycle; (2) propionyl-CoA carboxylase converts propionyl-CoA to methylmalonyl-CoA for the vitamin B12-depedent methylmalonyl pathway, which is used for for succinyl-Coa production; (3) propionyl-CoA, in the form of methylmalonyl-CoA, is used to generate building blocks for the *M. tb* cell wall [34]. Hence, 3HPA is a metabolite of the host, but also has strong association with altered gut microbiota metabolism; our study and previous reports indicate a clear link to *M. tb* metabolism.

### Prolonged immune response-related protein catabolism to treatment and induced *M. tb* death

IG, 3MCG and 2MBG were significantly elevated at T1 in TBM stages 2 and 3 (Fig. 3). In Fig. 4 it can be seen that for TBM stage 1, both 3MCG and 2MBG remained above that of the controls at a constant level (i.e., no trend), whereas IG for TBM stage 1 inexplicably increased during the course of treatment. For all three of these metabolites (IG, 3MCG and 2MBG), there was a clear linear trend of being significantly elevated at T1 and returning to control concentration levels by T6, for TBM stage 2 and 3. Loots et al. [35] also reported elevated urinary concentrations of 2MBG, and other organic acids associated with a multiple acyl-CoA dehydrogenase defect (MADD) metabolic profile in the urine of TB patients, supporting the result in this study. Chronic infection has been shown to cause vulnerability of the host, thus creating a cycle for survival, thereby activating specific metabolic pathways resulting in hypermetabolism, increased nitrogen loss, and enhanced gluconeogenesis. *M. tb* largely depends on human-derived nutrients for survival and replication including a source of nitrogen. Amino acids, including the BCAA amino acids isoleucine, leucine, and valine, are the most important organic nitrogen suppliers required by *M. tb* for intracellular growth, and survival against intracellular host-induced stress. All of the above contributes to TB cachexia, and subsequently, the WHO recommends, a patient with TB should consume more protein than is suggested for a healthy person (an additional 15–30% protein) [36–40]. Furthermore, 3MCG, IG and 2MBG can also be considered end products of β-oxidation of methyl branched-chain fatty acids of the *M. tb* cell wall, and the changes seen in our study can also be associated with *M. tb* death, or *M. tb* load.

### Differences in tryptophan metabolism between disease severity and time

5HIAA, a microbial catabolite of tryptophan, was significantly elevated in TBM stages 2 and 3 at T1, and returned close to control levels after 6 months of treatment. However, 5HIAA at TBM stage 1 remained elevated at a constant level throughout treatment. The tryptophan pathway has been indicated in studies as a key metabolic pathway in TBM [41, 42]. Tryptophan, a precursor used to synthesize the biogenic amine serotonin (5-hydroxytryptamine), is the main signalling molecule involved in several physiological processes and controls gut motility [43–45]. Serotonin, biosynthesized from tryptophan, functions as a neuromodulator and maintains homeostasis as a neuroendocrine and neurotransmitter [46]. Urinary excretion of 5HIAA serves as a marker for determining serotonin in neurological conditions, including Friedreich’s ataxia, schizophrenia, and olivo-ponto-cerebral atrophy [46–48]. The presence of 5HIAA in urine has been reported in a patient with carcinoid heart disease (CHD), with a high significant level as compared to patients without CHD. Kaltsas et al. [49] further confirmed that higher urine 5-HIAA levels are linked to an increased risk of developing CHD. Cheetham et al. [50] reported low levels of 5HIAA in cerebrospinal fluid of patients with depression, indicating increased serotonin breakdown. The biosynthesis of serotonin occurs in serotonergic neurons within the central nervous system in minor quantity, as well as in the enterochromaffin cells that lie along the gastrointestinal tract, where a majority is synthesized. 5HIAA is also a derivative of microbial indole-3-acetic acid and has been found to be a product of human gut microbiota, mostly synthesized in the kidney and liver, and passed in the urine [51, 52]. The microbe-produced indole derivatives of tryptophan have been shown to have a clear link to immune regulation in the host [53]. 5-Hydroxyindole (5HI), a microbial product and possible precursor of 5HIAA, is known to be involved in CNS regulation and antioxidant activity. 5HI protects cells by attenuating oxidative stress and consequently protects against mitochondrial dysfunction [54]. Liu et al. [55] shows that the microbial tryptophan metabolite 5HIAA can be used to assess intestinal homeostasis. Enrichment of microbial 5HIAA activates the Epac/Rap1 signalling pathway, which plays an important role in intestinal homeostasis Zhang et al. [56]. Furthermore, multi-omic profiling has shown that 5HIAA is a sensitive marker for intra-abdominal hypertension and sepsis in the host, and also that tryptophan-related metabolites (especially 5HIAA) are associated with microbiota changes, with several microbiota species correlating (positively and negatively) with 5HIAA [57]. Thus, 5HIAA is clearly a host metabolite, but is also a microbial metabolite, making it a co-metabolite. However, what is not clear from the literature is how 5HIAA is produced by gut microbiota. Moreover, a key question we ask is this: if increased flux of tryptophan into the kynurenine pathway is known to occur due to upregulated IDO-1 activity during TB, and other diseases [53, 58], then where/how is there sufficient bioavailability of tryptophan in the host for the increased levels of urinary 5HIAA that have been observed in this study?

### Disturbances in fatty acid metabolism

In our study, (5HHA) was significantly increased in TBM stages 2 and 3 at T1 and showed a decreasing linear trend that approached control levels for all three TBM stages by T6. 5HHA is a fatty acid dicarboxylic acid breakdown product that has been identified as a urinary microbial metabolite, under situations of fasting, aberrant fatty acid oxidation (FAO), and medium-chain triglyceride (MCT) feeding [59–61]. With the aim of elucidating mechanisms of TB, Luier and Loots [26] found anomalies in the host’s fatty acid and amino acid metabolism brought on by pulmonary TB. These fatty acids and degradation products comprised 5HHA, glycerol monostearate and 2-octenoic acid. Note that 2-octenedioic acid was significantly elevated in this study at T1, T3, T4, T5 and T6. Other fatty acids that arose at later treatment times as significant in this study were pimelic acid and suberic acid. It is known that *M. tb* uses fatty acids as primary sources of energy results; hence increased urinary fatty acids and their catabolic products are common in TB cases. Additionally, other factors that might influence the host’s response are different bacterial processes, which could help to elucidate the associated loss of weight in TB cases [26, 62, 63]. Hence, disturbances in fatty acid metabolism are another landmark of gut dysbiosis induced by TB. However, to our knowledge, 5HAA has not been directly linked to changes in gut microbiota.

### Perturbed tyrosine–phenylalanine metabolism

Lastly, 4HHA was significantly increased in TBM stages 2 and 3 at T1, and PAG was significantly increased only in TBM stage 1 at T1. Both metabolites showed similar linear trends – TBM stage 1 stayed elevated throughout treatment, whereas TBM stages 2 and 3 showed a decreasing trend, approaching control levels by T6. 4HHA and PA are components of host tyrosine–phenylalanine (aromatic amino acids) metabolism, and metabolites of microbial origin. 4HHA and PA have been identified as a major consequence of perturbed metabolism associated with diseases linked with alterations to the gut-microbiome [8, 40, 64–66]. A study by Mason et al. [8] examined the urinary profiles of (untreated) infants and children (≤ 13 years) from cohorts of 12 confirmed TBM cases, 12 suspected TBM cases that were later confirmed negative for TBM, and 29 healthy controls. Mason et al. [8] identified four metabolites, including 4HHA, as having potential for a non-invasive diagnostic biosignature, as well as an increase in PAG. In another study, Das et al. [67] compared the urinary metabolic profiles of patients with active pulmonary TB (*n* = 11) under treatment to those of healthy controls (*n* = 11). The urine metabolome of follow-up samples revealed a treatment-dependent trend for numerous metabolites, notably 4HHA; those who were deemed clinically healed displayed a metabolic profile comparable to that of asymptomatic healthy individuals, suggesting that a possible target for TB diagnosis and treatment is revealed by disrupted tyrosine–phenylalanine metabolism [67]. Children with autism have also been found to produce abnormal PA excretion through the urine [68]. PA has also been linked to liver dysfunction and this aligns with the pathogenesis of TBM; namely, by TBM stage 3 the patient has had the infection for two or more weeks and the accumulated hepatotoxins have been responsible for some damage to the liver. This could explain why only TBM stage 3 cases showed significantly increase PA in our study.

### Urinary biomarkers for *M. tb*

Of note, methylcitric acid (MCA) was identified as significant at T3, T4 and T5, and methylsuccinic acid (MSA) was observed as significant at T5 and T6. MCA and MSA have been linked to *M. tb* metabolism by several studies [8, 69–72]. Therefore, we postulate that some of the TBM patients retained a reservoir of *M. tb* somewhere in their body – that is, *M. tb* was not entirely eradicated by treatment. This is an untested postulate, but one that we would like to test in future treatment studies.

### Limitations of this study

Previous work has shown that tuberculostatic antibiotics cause changes in the gut microbiota community, and associated metabolism [73, 74]. These findings have not escaped our attention. It should be stated that we cannot definitively demarcate which metabolites are strictly the response of treatment or disease. Treatment invariably has some effect(s). For our study, however, we focused on eight altered metabolites that we consider to be most closely associated with the disease – TBM, and its associated dysbiosis. Other limitations of this study are that follow-up *M. tb* tests were not done and there are no data on the status of the TBM patients after treatment. Finally, the time of baseline sample (T1), collected upon release from hospital, was variably different for each TBM patient (an uncontrolled confounder). Each TBM patient therefore required a unique amount of time to stabilize in hospital before being discharged.

## Conclusions

We identified 2-methylbutyrlglycine, 3-hydroxypropionic acid, 3-methylcrotonylglycine, 4-hydroxyhippuric acid, 5-hydroxyindoleacetic acid, 5-hydroxyhexanoic acid, isobutyrylglycine, and phenylacetylglutamine as urinary markers of dysbiosis in TBM. These urinary metabolites can be associated with host and/or microbial metabolism and appear to be fundamental to TB. This study lays the foundation for identifying products of microbial metabolites as novel points for management of TBM treatment. It is recommended that further studies be conducted to identify the altered gut microbiota linked to TBM pathogenesis and treatment efficacy. We recommend also that future studies employ both metabolomics and metagenomics on fecal samples collected from TBM cases to properly assess the gut microbiome.

### Electronic supplementary material

Below is the link to the electronic supplementary material.

Supplementary Material 1

## Data Availability

The entire set of LC/MS data generated during this metabolomics research will be made available to the public in 2024, once the project is completed and all the data are published. On special request, immediate subsets of data from the study published here can be made accessible. (Email: nmr.nwu@gmail.com)
