# Supplementary material for: Urinary markers of Mycobacterium tuberculosis and dysbiosis in paediatric tuberculous meningitis cases undergoing treatment
Source: Gut Pathog. 2024 Mar 12;16:14. doi: 10.1186/s13099-024-00609-9 (PMC10936073; doi:10.1186/s13099-024-00609-9)
Supplement: Supplementary file 1 — Supplementary Material 1 [file 13099_2024_609_MOESM1_ESM.docx]

**Supplementary Information: Urinary markers of *Mycobacterium tuberculosis* and dysbiosis in paediatric tuberculous meningitis cases undergoing treatment**

**Simon Isaiah^1^, Du Toit Loots^1^, A. Marceline Tutu van Furth^2^, Elmarie Davoren^3^, Sabine van Elsland^4,5^, Regan Solomons^5^, Martijn van der Kuip^2#^, Shayne Mason^1*#^**

^1^ Human Metabolomics, Faculty of Natural and Agricultural Sciences, North-West University, Potchefstroom, South Africa.

^2^ Vrije Universiteit, Pediatric Infectious Diseases and Immunology, Amsterdam University Medical Centers, Emma Children’s Hospital, De Boelelaan 1117, Amsterdam, The Netherlands.

^3^ Centre for Human Metabolomics, North-West University, Potchefstroom, South Africa.

^4^ MRC Centre for Global Infectious Disease Analysis, Imperial College London, United Kingdom.

^5^ Department of Paediatrics and Child Health, Faculty of Medicine and Health Sciences, Stellenbosch University, Cape Town, South Africa.

***^#^****Shared last authorship*

********Corresponding author: Shayne Mason;* [*nmr.nwu@gmail.com*](mailto:nmr.nwu@gmail.com)*; Tel.: +27 (18) 285 2532*


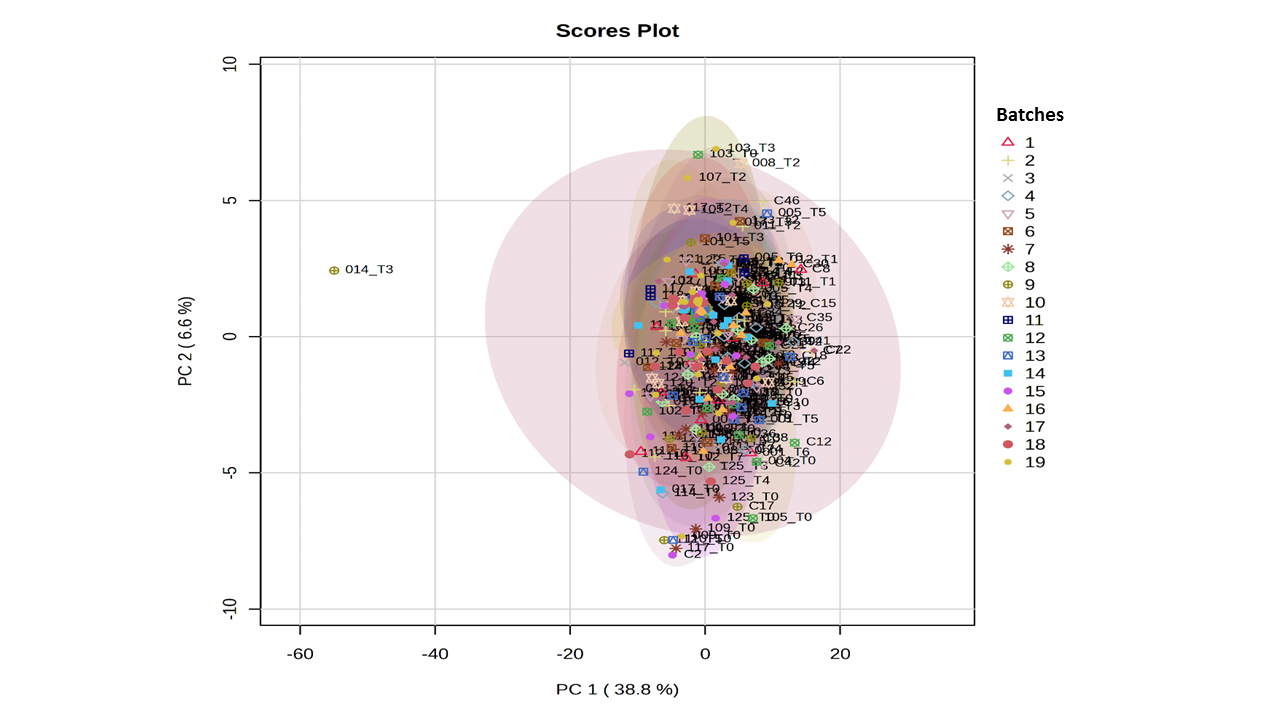


Fig S1: A PCA of all the batches (n=19). Notably, one case (014_T3) stood out as an outlier, and was removed from analysis.


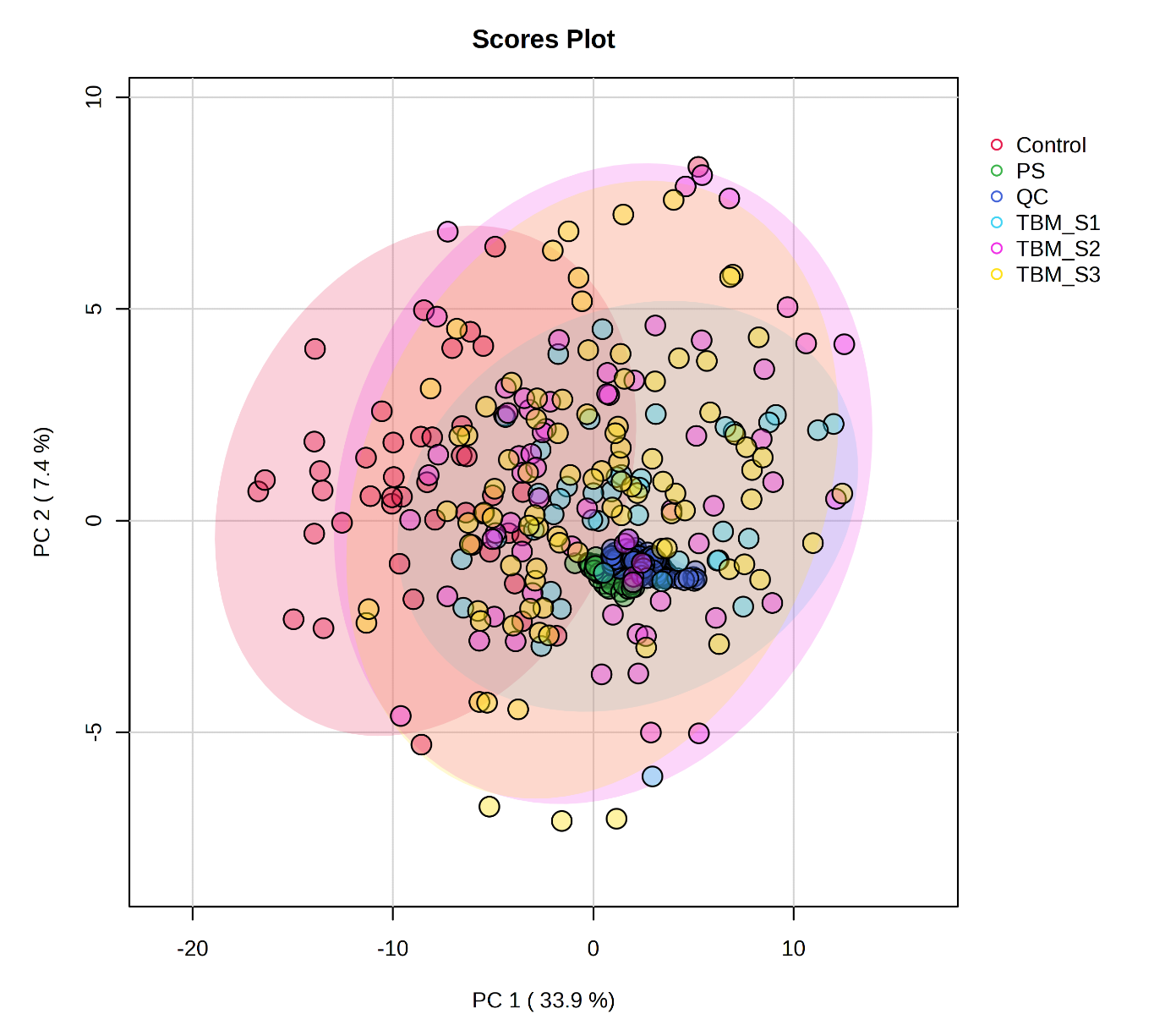


Fig S2: PCA showing that the samples of the QC (dark blue; pooled quality control urine sample) and PS (green; pooled patient urine sample) clustered closely together; supporting the conclusion that there was no batch effect from the analytical instrument.


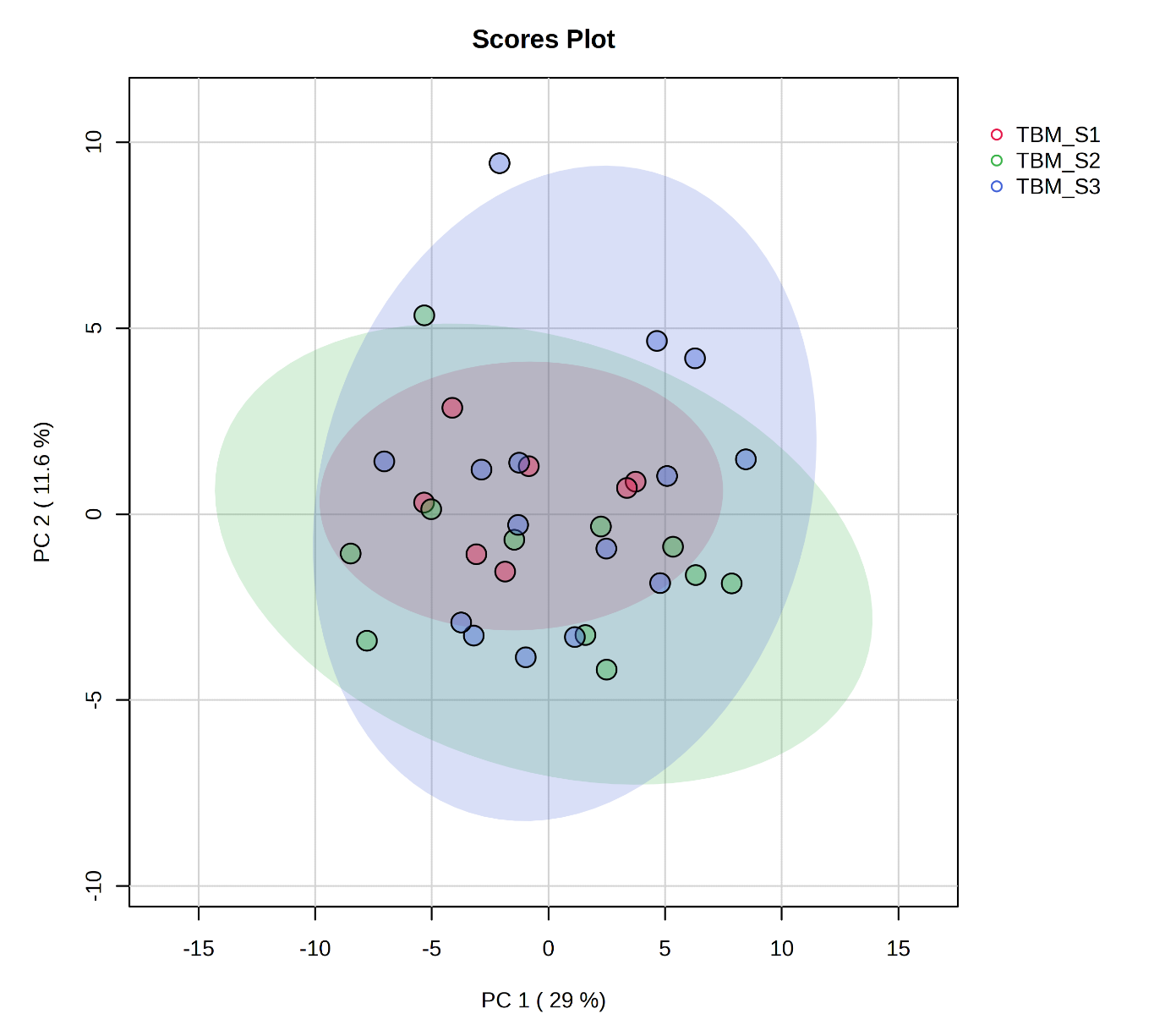


Fig S3: PCA of the three TBM stages at T1 shows no characteristic grouping nor differentiation between the stages.

Table S1: Demographics of experimental study cohort affirmed as definite TBM, organized into stages (1, 2 and 3).

| **CRITERIA** | **TBM 1** | **TBM 2** | **TBM 3** |
| --- | --- | --- | --- |
|  | **n/N (%)** | **n/N (%)** | **n /N (%)** |
| Male gender | 4/8 (50) | 7/11 (63.6) | 9/16 (56.3) |
| Age (months) (Median [IQR]) | 65 [30 – 117] | 46 [10 – 135] | 43 [22 – 158] |
| **Clinical symptoms** |  |  |  |
| Fever | 4/8 (50) | 5/11 (45.5) | 14/16 (87.5) |
| Night sweats | 0 | 2/11 (18.2) | 2/16 (12.5) |
| Poor feeding | 2/8 (25) | 2/11 (18.2) | 5/16 (31.3) |
| Weight loss | 3/8 (35.7) | 2/11 (18.2) | 5/16 (31.3) |
| Vomiting without diarrhoea | 3/8 (35.7) | 2/11 (18.2) | 4/16 (25) |
| Persistent coughing >14d | 1/8 (12.5) | 0 | 1/16 (6.3) |
| Headache | 2/8 (25) | 3/11 (27.3) | 1/16 (6.3) |
| Seizures | 3/8 (35.7) | 1/11 (9.1) | 5/16 (31.3) |
| Lethargy | 2/8 (25) | 2/11 (18.2) | 2/16 (12.5) |
| **Neurological signs** |  |  |  |
| GCS: (median [IQR]) | 15 [15] | 14.5 [12 – 15] | 10.5 [7 – 15] |
| Meningism | 0 | 4/11 (36.4) | 3/16 (18.7) |
| Focal motor deficit | 0 | 2/11 (18.2) | 4/16 (25) |
| Cranial nerve palsy | 0 | 1/11 (9.1) | 3/16 (18.7) |
| Raised ICP | 0 | 2/11 (18.2) | 5/16 (31.3) |
| **Neuroimaging (CT brain)** |  |  |  |
| Hydrocephalus | 2/8 (25) | 6/11 (54.5) | 11/16 (68.7) |
| Infarctions | 0 | 2/11 (18.2) | 1/16 (6.3) |
| Tuberculoma | 2/8 (25) | 1/11 (9.1) | 3/16 (18.7) |
| Meningeal enhancement | 2/8 (25) | 2/11 (18.2) | 8/16 (50) |
| VP shunt present | 0 | 1/11 (9.1) | 4/16 (25) |
|  |  |  |  |
| **Other radiology** |  |  |  |
| CXR (signs of pulmonary TB) | 1/8 (12.5) | 3/11 (27.3) | 2/16 (12.5) |
| **Laboratory values** |  |  |  |
| Blood sodium (mmol/L) (median [IQR]) | 139 [130 – 139] | 136 [135 – 139] | 131.5 [126 – 141] |
| Blood total protein (g/L) (median [IQR]) | 68 [68] | 74 [73 – 75] | 78.5 [75 – 82] |
| Blood glucose (mmol/L) (median [IQR]) | 0 | 5.9 [0 – 5.9] | 7.1 [4.9 – 7.7] |
| Blood lipids (median [IQR]) | 0 | 0 | 3.9 [0 – 3.9] |
| CSF protein (g/L) (median [IQR]) | 0.12 [0.12 – 0.53] | 0.94 [0.87 – 2.1] | 0.84 [0.78 – 2.73] |
| CSF glucose (mmol/L) (median [IQR]) | 2.6 [2.6 – 3.6] | 2.05 [1.4 – 2.7] | 1.8 [0.67 – 2.82] |
| CSF lymphocytes (cells/µL) (median [IQR]) | 0 [0 – 40] | 157 [103.5 – 346.7] | 57 [40 – 80] |
| CSF lymphocytes (%) (median [IQR]) | 0 [0 – 43.5] | 92.2 [78.9 – 93.18] | 95.6 [94.5 – 100] |
| HIV | 0 | 0 | 0 |
| Key: IQR = Interquartile range, CXR = Chest X-ray, GCS= Glasgow coma scale, CSF = Celebrospinal fluid, ICP = Intracranial pressure, VP = Ventriculoperitoneal, HIV = human immunodeficiency virus. | | | |
|  | | | |

Table S2: Quantitative data indicating 30 metabolites at T1 that are of statistical and practical importance [PLS-DA VIP value (component 1 AND 2) >1.0 AND ANOVA Kruskal-Wallis adjusted p-value < 0.01 AND effect size d-value > 0.8]. For Effect size conditional highlighting: d < 0.8 = red dot; 0.8 ≤ d ≤ 1.0 = yellow dot; d > 1.0 = green dot.

Table S3: Seventy-eight (78) targeted compounds of interest used in this study, including standards/isotopes.

| **Compounds of interest including internal standards/isotopes** | **Type** | **Distributer** | **Purity** | **CAS no** | **Product code** |
| --- | --- | --- | --- | --- | --- |
| 1,3-Dimethyluric acid | Standard | ChemCruz |  |  | sc- |
| 2,3-Pyridinedicarboxylic acid | Standard | Aldrich | 99% | 89-00-9 | P63204-25G |
| 2,5‐Furandicarboxylic acid | Standard | Sigma | 97% | 3238-40-2 | 722081-5G |
| 2-Furoylglycine | Standard | Sigma | 98% | 5657-19-2 | SMB00966 |
| 2-Hydroxy-3-Methylbutyric acid | Standard | Aldrich | 99% | 4026-18-0 | 219835-1G |
| 2-Hydroxyadipic acid | Standard | Sigma | 95% |  | 92826-10MG |
| 2-Hydroxydecanedioic acid | Standard | Industrial Analytical | 98% | 103963-71-9 | H939450 |
| 2‐Hydroxyglutaric acid disodium salt | Standard | Sigma | 98% | 63512-50-5 | 61313-10MG |
| 2-Hydroxymethylbutyric acid | Standard | Sigma |  |  | 96045-10MG |
| 2‐Hydroxyphenylacetic acid | Standard | Aldrich | 99% | 614-75-5 | H49804-10G |
| 2-Ketoglutaric acid disodium salt hydrate | Standard | Sigma | 95% | 305-72-6 | K3752-5G |
| 2‐Methylbutyrylglycine | Standard | VUMC - Organic Synthesis Laboratory | 95% |  |  |
| 2-Octenedioic acid | Standard | Sigma | 95% | 124791-62-4 | 92675-10MG |
| 3-(3,5-Dihydroxyphenyl) propanoic acid | Standard | Industrial Analytical | |  | TRCD267960 |
| 3-(3-Hydroxyphenyl)-3-Hydroxypropionic acid (HPHPA) | Standard | Sigma | 95% | 3247-75-4 | 06704-10MG |
| 3-(3-Hydroxyphenyl)-propionic acid | Standard | Sigma |  | 621-54-5 | 91779-50MG |
| 3,4-Dihydroxybenzoic acid | Standard | Sigma | 97% | 99-50-3 | 08992-50MG |
| 3,4‐Dihydroxyphenylacetic acid (L‐DOPAC) | Standard | Industrial Analytical | 98% | 102-32-9 | D454250 |
| 3‐Hydroxy‐2‐methylbutyric acid (HMBA) | Standard | Industrial Analytical | 95% |  | H946485 |
| 3‐Hydroxybutyric acid (DL-) sodium salt | Standard | Sigma | 98% | 150-83-4 | H6501-5G |
| 3-Hydroxydodecanedioic acid | Standard | Industrial Analytical | 93% | 34574-69-1 | H939615 |
| 3‐Hydroxyglutaric acid | Standard | Sigma | 95% | 638-18-6 | 04725-10MG |
| 3-Hydroxyisobutyrate sodium | Standard | Sigma | 96% | 1228078-57-6 | 11161-100MG |
| 3‐Hydroxyisovaleric acid/3-Hydroxy-3-methylbutyric acid | Standard | Sigma | 95% | 625-08-1 | 55453-1G |
| 3‐Hydroxypropionic acid | Standard | Sigma |  | 503-66-2 | 792659-1G |
| 3‐Hydroxysebacic acid | Standard | Industrial Analytical | 97% | 73141-46-5 | H953770 |
| 3‐Indoleacetic acid | Standard | Aldrich | 98% | 87-51-1 | I3750-5G-A |
| 3‐Methylcrotonylglycine | Standard | VUMC - Organic Synthesis Laboratory | 95% |  |  |
| 3‐Methylglutaconic acid | Standard | Fluka | 97% | 372-42-9 | 44108-10MG |
| 3‐Phenyllactic acid | Standard | Aldrich | 98% | 828-01-3 | P7251-1G |
| 4‐Hydroxyhippuric acid | Standard | Industrial Analytical | 95% | 2482-25-9 | H827810 |
| 4‐Hydroxyphenylacetic acid | Standard | Sigma | 98% | 156-38-7 | 55206-10MG |
| 4‐Hydroxyphenyllactic acid (DL-) | Standard | Aldrich | 97% | 6482-98-0 | H3253-500MG |
| 4‐Pyridoxic acid | Standard | Aldrich | 98% | 82-82-6 | P9630-25MG |
| 5-Hydroxyhexanoic acid | Standard | ChemCruz |  |  | sc-352672 |
| 5‐Hydroxyindoleacetic acid (5‐HIAA) | Standard | Sigma | 98% | 54-16-0 | H8876-100MG |
| 5-Hydroxymethyl-2-furancarboxylic acid | Standard | Sigma | 95% | 6338-41-6 | 902845-50MG |
| Acetylaspartic acid | Standard | Sigma | 99% | 997-55-7 | 00920-5G |
| Aconitic acid | Standard | Aldrich | 98% | 4023-65-8 | 122750-25G |
| Azeliac acid | Standard | Sigma | 99% | 123-99-9 | 95054-100MG |
| Benzoic acid | Standard | Sigma | 99% | 65-85-0 | 242381-100G |
| Butyrylglycine | Standard | VUMC - Organic Synthesis Laboratory | 95% |  |  |
| Citric acid | Standard | Sigma | 99% | 77-92-9 | C0759-500G |
| Ethylmalonic acid | Standard | Aldrich | 97% | 601-75-2 | 102687-5G |
| Glucaric acid (D-Saccharic acid potassium salt) | Standard | Sigma | 98% | 576-42-1 | S4140-10G |
| Glutaric acid | Standard | Aldrich | 99% | 110-94-1 | G3407-25G |
| Heptanoylglycine | Internal Standard | Sigma | Analytical std | 23783-23-5 | 93539-50MG |
| Hexanoylglycine | Standard | VUMC - Organic Synthesis Laboratory | 95% |  |  |
| Hippuric acid | Standard | Sigma | 98% | 495-69-2 | 112003-100G |
| Homogentisic acid | Standard | Sigma | 98% | 451-13-8 | H0751-100MG |
| Homovanillic acid (HVA) | Standard | Sigma | 98% | 306-08-1 | H1252-250MG |
| Hydantoin‐5‐propionic acid/3-(2,5-Dioxoimidazolidin-4-yl) propionic acid | Standard | Industrial Analytical | 95% | 5624-26-0 | D486445 |
| Isobutyrylglycine | Standard | VUMC - Organic Synthesis Laboratory | 95% |  |  |
| Isocitric acid (trisodium salt hydrate, DL-) | Standard | Sigma | 93% | 1637-73-6 | I1252-1G |
| Isovalerylglycine | Standard | VUMC - Organic Synthesis Laboratory | 95% |  |  |
| Kynurenic acid | Standard | Sigma | 98% | 492-27-3 | K3375-1G |
| Malic acid | Standard | Aldrich | 98% | 6915-15-7 | M0875-100G |
| Methylcitric acid/2-Methylcitric acid | Standard | Industrial Analytical | 97% | 6061-96-7 | TRCM265080 |
| Methylmalonic acid | Standard | Aldrich | 99% | 516-05-2 | M54058-25G |
| Methylsuccinic acid | Standard | Sigma | 99% | 3641-51-8 | 415596-5G |
| Methyluric acid | Standard | ChemCruz |  |  | sc-208662 |
| Methylxanthine | Standard | Sigma | 97% | 6136-37-4 | 69720-250MG |
| N-Acetylphenylalanine | Standard | Aldrich | 99% | 2018-61-3 | 857459-1G |
| N‐acetyltyrosine | Standard | Sigma | 100% | 537-55-3 | PHR1173-1G |
| Phenylacetyl-L-glutamine | Standard | Sigma | 95% | 28047-15-6 | SMB00962-25MG |
| Pimelic acid | Standard | Aldrich | 98% | 111-16-0 | P45001-25G |
| Propionylglycine | Standard | VUMC - Organic Synthesis Laboratory | 95% |  |  |
| Pyroglutamic acid (5‐oxoproline) | Standard | Aldrich | 99% | 98-79-3 | 83160-25G |
| Sebacic acid | Standard | Aldrich | 99% | 111-20-6 | 283258-5G |
| Suberic acid | Standard | Aldrich | 98% | 505-48-6 | S5200-5G |
| Suberylglycine | Standard | VUMC - Organic Synthesis Laboratory | 95% |  |  |
| Succinic acid | Standard | Sigma | 99% | 110-15-6 | S3674-100G |
| Tiglylglycine | Standard | VUMC - Organic Synthesis Laboratory | 95% |  |  |
| Tricarballylic acid | Standard | Aldrich | 99% | 99-14-9 | T53503-25G |
| Uracil | Standard | Sigma | 99% | 66-22-8 | U0750-5G |
| Vanillactic acid (DL-Vanillactic acid lithium salt hydrate ) | Standard | Sigma | 97% | 2475-56-1 (free acid) | 89127-50MG |
| Vanillic acid | Standard | Sigma | 97% | 121-34-6 | 94770-10G |
| Vanillylmandelic acid (VMA)/DL-4-Hydroxy-3-methoxymandelic acid | Standard | Sigma | 98% | 55-10-7 | H0131-100MG |
| Xanthurenic acid | Standard | Aldrich | 96% | 59-00-7 | D120804-1G |

Table S4: Multiple reaction monitoring (MRM’s) values and expected retention times of compounds of interest.

| **Name of compound** | **MRM** | **Expected RT*** |
| --- | --- | --- |
| 1,3-Dimethyluric acid | 195.1 -> 180.1 | 5.86 |
| 2 5 Furandicarboxylic acid | 155 -> 111.1 | 5.03 |
| 2 Furoylglycine | 168 -> 67.1 | 5.54 |
| 2 Hydroxy 3 Methylbutyric acid | 117 -> 71.2 | 5.73 |
| 2 Hydroxyadipic acid | 161 -> 71 | 4.14 |
| 2 Hydroxydecanedioic acid | 217.1 -> 155.2 | 10.19 |
| 2 Hydroxyglutaric acid | 147 -> 85.2 | 1.82 |
| 2 Hydroxymethylbutyric acid | 117 -> 87.2 | 5.41 |
| 2 Hydroxyphenylacetic acid | 151 -> 107 | 9.00 |
| 2 Ketoglutaric acid | 145 -> 101 | 1.37 |
| 2 Methylbutyrylglycine | 158 -> 74 | 6.30 |
| 2 Octenedioic acid | 171.1 -> 127.2 | 9.16 |
| 2,3-Pyridinecarboxylic acid | 166 -> 122 | 1.81 |
| 3 4 Dihydroxybenzoic acid | 153 -> 109.1 | 6.20 |
| 3 4 Dihydroxyphenylacetic acid | 167 -> 123.1 | 6.71 |
| 3 Hydroxy 2 Methylbutyric acid | 117 -> 73.1 | 4.82 |
| 3 Hydroxybutyric acid | 103 -> 59.2 | 2.83 |
| 3 Hydroxydodecanedioic acid | 245.1 -> 59.2 | 12.28 |
| 3 Hydroxyglutaric acid | 147 -> 85.1 | 2.16 |
| 3 Hydroxyisobutyric acid | 103 -> 73.2 | 3.17 |
| 3 Hydroxyisovaleric acid | 117 -> 59.2 | 4.74 |
| 3 Hydroxypropionic acid | 89 -> 59 | 1.29 |
| 3 Hydroxysebacic acid | 217.1 -> 59.1 | 9.51 |
| 3 Indoleacetic acid | 174.1 -> 130.1 | 11.61 |
| 3 Methylcrotonylglycine | 156.1 -> 74.1 | 6.62 |
| 3 Methylglutaconic acid | 143 -> 99.1 | 6.66 |
| 3 Phenyllactic acid | 165.1 -> 119.1 | 6.48 |
| 3-(3,5-Dihydroxyphenyl)propionic acid | 181.1 -> 137.1 | 7.40 |
| 3-(3-Hydroxyphenyl)-3-Hydroxypropionic acid | 181.1 -> 121.1 | 7.00 |
| 3-(3-Hydroxyphenyl)-propionic acid | 165.1 -> 119.1 | 9.89 |
| 4 Hydroxy 3 Methoxymandelic acid | 197 -> 137 | 4.65 |
| 4 Hydroxyphenylacetic acid | 151 -> 107.1 | 7.91 |
| 4 Hydroxyphenyllactic acid | 181.1 -> 135.1 | 6.83 |
| 4 Pyridoxic acid | 182 -> 108 | 4.73 |
| 4-Hydroxyhippuricacid | 194 -> 100.1 | 6.28 |
| 5 Hydroxyindoleacetic acid | 190.1 -> 146.1 | 7.80 |
| 5-Hydroxyhexanoic acid | 131 -> 85.2 | 6.18 |
| 5-Hydroxymethyl-2-Furancarboxylic acid | 141 -> 97 | 5.30 |
| Aconitic acid | 173 -> 129.2 | 2.90 |
| Azeliac acid | 187.1 -> 125.1 | 11.23 |
| Benzoic acid | 121 -> 77.2 | 10.80 |
| Buterylglycine | 144 -> 74.2 | 4.88 |
| Citric acid | 191 -> 111 | 2.28 |
| Ethylmalonic acid | 131 -> 87.1 | 5.43 |
| Glucaric acid | 209 -> 191 | 0.79 |
| Glutaric acid | 131 -> 87.2 | 4.75 |
| Heptanoylglycine (IS) | 186 -> 74.2 | 11.70 |
| Hexanoylglycine | 172.1 -> 74.1 | 9.42 |
| Hippuric acid | 178.1 -> 134.1 | 7.80 |
| Homogentisic acid | 167 -> 122.1 | 5.49 |
| Homovanillic acid | 181.1 -> 137.1 | 8.49 |
| Hydantoin 5 propionic acid | 171 -> 100.1 | 3.48 |
| Isobutyrylglycine | 144.1 -> 74.2 | 4.67 |
| Isocitric acid | 191 -> 111 | 1.18 |
| Isovalerylglycine | 158.1 -> 74.1 | 6.58 |
| Kynurenic acid | 188 -> 144.1 | 7.29 |
| Malic acid | 133 -> 115 | 1.13 |
| Methylcitric acid | 205 -> 125.1 | 4.62 |
| Methylmalonic acid | 117 -> 73.2 | 3.08 |
| Methylsuccinic acid | 131 -> 87.1 | 5.42 |
| Methyluric acid | 181 -> 138.1 | 5.01 |
| Methylxanthine | 165 -> 80.2 | 5.34 |
| N Acetylasparitc acid | 174 -> 88.1 | 1.67 |
| N Acetylphenylalanine | 206.1 -> 91.2 | 9.73 |
| N Acetyltyrosine | 222.1 -> 178.1 | 7.07 |
| Phenylacetylglutamine | 263.1 -> 127.1 | 7.90 |
| Pimelic acid | 159.1 -> 97.2 | 7.96 |
| Propionylglycine | 130 -> 74.2 | 2.58 |
| Pyroglutamic acid (Positive mode) | 130 -> 84.1 | 2.27 |
| Sebacic acid | 201.1 -> 183.2 | 12.75 |
| Suberic acid | 173.1 -> 111.2 | 9.63 |
| Suberylglycine | 230.1 -> 212.3 | 7.92 |
| Succinic acid | 117 -> 73 | 2.80 |
| Tiglylglycine | 156.1 -> 112.1 | 6.46 |
| Tricarballylic acid | 175 -> 69.2 | 3.40 |
| Uracil | 111 -> 42.3 | 1.65 |
| Vanillactic acid | 211.1 -> 193.3 | 7.52 |
| Vanillic acid | 167 -> 152.1 | 8.16 |
| Xanthurenic acid | 204 -> 160.2 | 6.92 |

Table S5: Binary gradient conditions and experimental parameters of LC-MS/MS.

| **Time** | **Mobile Phase 1  (H2O with 0.1% Formic acid)** | **Mobile Phase 2  (Acetonitrile with 0.1% Formic acid)** |
| --- | --- | --- |
| 0.00 min | 100% | 0 |
| 1.50 min | 100% | 0 |
| 2 min | 98% | 2% |
| 2.5 min | 95% | 5% |
| 3.5 min | 93% | 7% |
| 6 min | 85% | 15% |
| 7.5 min | 80% | 20% |
| 9 min | 75% | 25% |
| 10.5 min | 70% | 30% |
| 12 min | 60% | 40% |
| 15 min | 25% | 75% |
| 17 min | 5% | 95% |
| 22 min | 100% | 0 |
| 25 min | 100% | 0 |
|  |  |  |
| **Agilent Jetstream ESI Source Parameters** | |  |
| Gas Temp | 170⁰C |  |
| Gas Flow | 10 l/min |  |
| Nebulizer | 25 psi |  |
| Sheath Gas Temp | 350⁰C |  |
| Sheath Gas Flow | 12 l/min |  |
| Capillary | 3000 V Negative |  |
| Nozzle Voltage | 500 V |  |
